# Supplementary material for: The genome sequence of the Dotted Grey Groundling, Athrips mouffetella (Linnaeus, 1758)
Source: Wellcome Open Res. 2024 Feb 19;9:42. [Version 1] doi: 10.12688/wellcomeopenres.20840.1 (PMC11249505; doi:10.12688/wellcomeopenres.20840.1)
Supplement: Peer review history

# Reviewer report: Reviewer response for version 1

**DOI:** 10.21956/wellcomeopenres.23059.r82148

**Published:** 2024-07-15

**Authors:** Paul Mireji

**Affiliations:** 1 Biotechnology Research Institute, Kenya Agricultural and Livestock Research Organization, Kikuyu, Kenya

In this manuscript, the authors sequenced and annotated the genome of Dotted Grey Groundling, from UK part of the part of the Darwin Tree of Life Project focused on sequencing all named eukaryotic species in Britain and Ireland. The authors employed standard/established procedures and protocols in this excise. The findings/conclusions are acceptable and supported by the data. It appears that the focus of the project, at this point on generating generic/automatic annotations that can be refined later thorough community annotations, probably by end users. This might account for absence of in-depth information on the annotations, such as gene families identified, which should have come out clearly. That being the case, I have the following minor concerns.

1. The authors should provide more information on the Darwin Tree of Life Project and probably what informed the choice of this species for preferential sequencing/annotations beyond the goal of the project and feeding on honeysuckle and snowberry.
2. Better description of identification/validation of the specimen e.g. taxonomic key other that who and where this was done [identified by Douglas Boyes (University of Oxford)] to facilitate reproducibility.   

Are sufficient details of methods and materials provided to allow replication by others?

Yes

Is the rationale for creating the dataset(s) clearly described?

Partly

Are the datasets clearly presented in a useable and accessible format?

Yes

Are the protocols appropriate and is the work technically sound?

Yes

Reviewer Expertise:

Medical Entomology and genomics

I confirm that I have read this submission and believe that I have an appropriate level of expertise to confirm that it is of an acceptable scientific standard.

# Reviewer report: Reviewer response for version 1

**DOI:** 10.21956/wellcomeopenres.23059.r87328

**Published:** 2024-07-15

**Authors:** Melody Clark

**Affiliations:** 1 Natural Environment Research Council, Cambridge, UK

This article describes the genome sequencing of the Dotted Grey Groundling, a moth common to the southern two thirds of England. The sequenced specimen was sourced from Wytham Woods. This is one of the most researched areas of woodland in the world and includes a Wytham Genome Project which aims to sequence all species in this wood. The Wytham Genome Project is a key site for the Darwin Tree of Life Project. The protocols underpinning the genome sequencing and assembly are fully described and referenced. The bioinformatic tools used to assemble, curate and annotate this genome are all freely available. The genome assembly is of very high quality with most data scaffolded to 31 chromosomal pseudomolecules, including the sex chromosomes. These data are freely available for reuse.

Are sufficient details of methods and materials provided to allow replication by others?

Yes

Is the rationale for creating the dataset(s) clearly described?

Yes

Are the datasets clearly presented in a useable and accessible format?

Yes

Are the protocols appropriate and is the work technically sound?

Yes

Reviewer Expertise:

Genomics, transcriptomics, organisms responses to change

I confirm that I have read this submission and believe that I have an appropriate level of expertise to confirm that it is of an acceptable scientific standard.
